# Trypanosome infection rate in *Glossina tachinoides*: infested rivers of Limmu Kosa District Jimma Zone, Western Ethiopia

**DOI:** 10.1186/s13104-020-04970-1

**Published:** 2020-03-05

**Authors:** Behablom Meharenet, Dereje Alemu

**Affiliations:** 1 National Institute for Control and Eradication of Tsetse Fly and Trypanosomosis, Kaliti Tsetse Fly Mass Rearing and Irradiation Center, P.O. Box: 19917 Addis Ababa, Ethiopia; 2 Bedele Tsetse Fly and Trypanosomosis Control and Investigation Center, Bedele, Ethiopia

**Keywords:** Limmu Kosa District, Trypanosome, Infection rate, *Glossina tachinoides*

## Abstract

**Objective:**

Trypanosomosis is a disease of domestic animals and humans resulting from infection with parasitaemic protozoa of the genus *Trypanosoma* transmitted primarily by tsetse flies. A cross-sectional study was conducted from January-March 2018, to estimate the infection rate of trypanosome in *Glossina tachinoides*, their distribution, magnitude and involved trypanosome species in Limmu Kosa District of Jimma zone.

**Results:**

Study methodology involved entomological survey using monoconical traps to study the magnitude of Fly density Flay/Trap/Day (FTD) and tsetse fly dissection to estimate infection rate of trypanosome in vector flies. The study result indicated that there was only one species of Tsetse fly *Glossina tachinoides* detected with FTD = 4.45. From the total of (n = 284) dissected *Glossina tachinoides* flies only (n = 5) positive for Trypanosome resulting in 1.76% Infection Rate. Peak trypanosome infections were observed in female tsetse 2.04%, n = 4 and 1.14%, n = 1 in males. Furthermore, 1.06% of *Glossina tachinoides* were infected by *Trypanosome vivax* and the remaining 0.70% was *Trypanosome congolense*. Finally, the study concluded with the recommendation of control and suppression of the vector and parasite was mandatory due to Pathogenic Animal Trypanosomosis.

## Introduction

Trypanosomosis is a disease of domestic animals and humans resulting from infection with parasitaemic protozoa of the genus *Trypanosome* transmitted primarily by the tsetse flies (*Glossina species*). Trypanosome parasitizes all classes of vertebrates including human beings and it is predominantly a parasite of blood [1]. Its prevalence and infection rate depends on the vectorial capacity of *Glossina* species responsible for transmission. From the three groups (based on habitat) of *Glossina*, the savannah and Palpalis species are the most important since they inhabit areas suitable for grazing and watering lands of animal production [2]. Hence, the objective of this research was to estimate the infection rate of trypanosome in *Glossina tachinoides*, their distribution, magnitude and involved trypanosome species in Limmu Kosa District of Jimma zone.

## Main text

### Materials and methods

#### Description of the study area

**Fig. 1:**
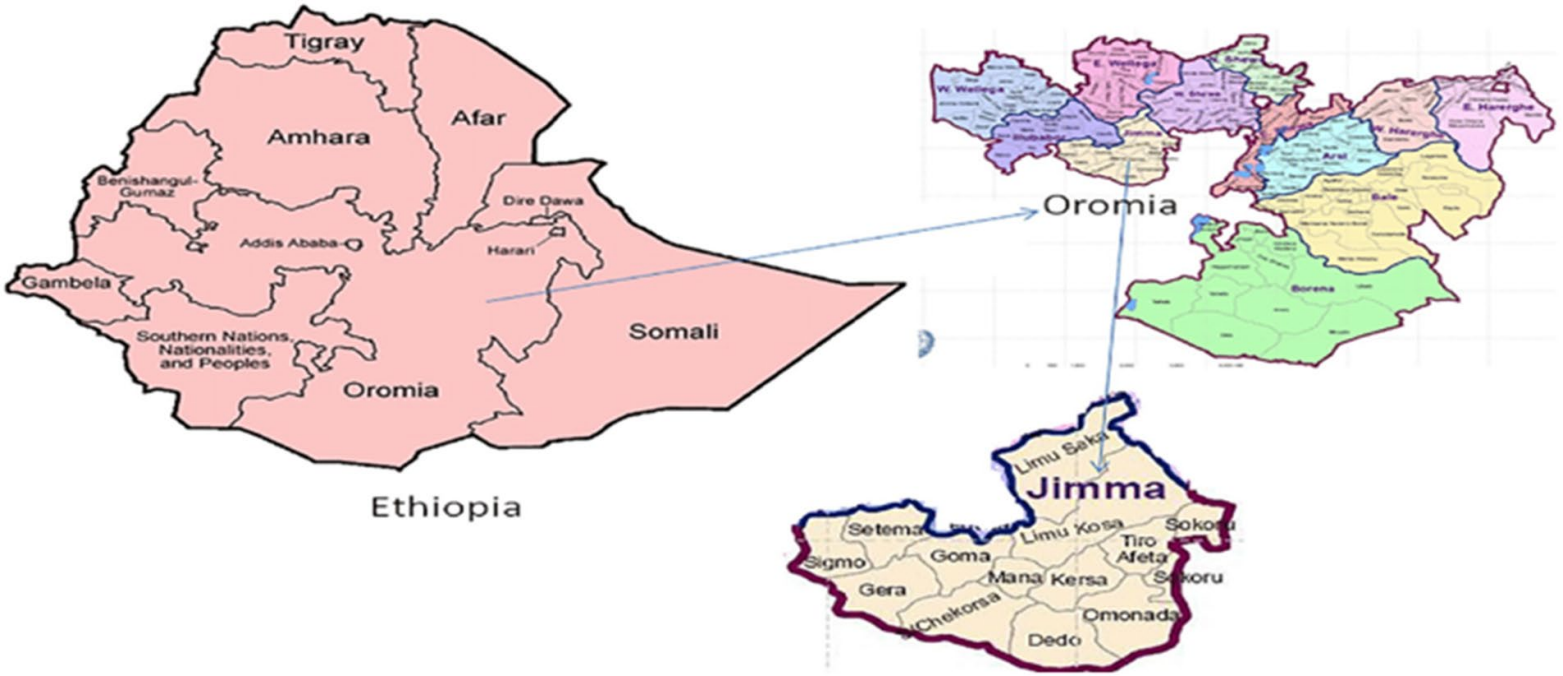
Map of the study area cited from Malaria journal, vol-18 issue-(1), p. 236

Limmu Kosa is one of the districts located in the Oromia Regional State of Ethiopia Jimma Zone. It is bordered on the South by Kersa, on the Southwest by Mana, on the West by Gomma, on the Northwest by the Didessa River which separates it from the Illubabor Zone, on the north by Limmu Sekka, on the Northeast by the Gibe River which separates it from the West Shewa Zone and the Southern Nations, Nationalities and Peoples Region, on the East by Sokoru, and on the Southeast by Tiro Afeta. The District was located in the altitude ranging between 1200 and 3020 meters above sea level. The rivers located in the district include the Didesa main river basin, Awetu and the Dembi which are tributaries of the main river basin [3, 4] (Fig. 1).

### Study design and methodologies

#### Entomological study

A total of 70 monoconical standard traps were deployed in the main Didessa River and most tributaries located in four different peasant associations namely Adis limat, Burqa gudina, Gale jimate and Busase with octenol (1-oct-3-nel), acetone and 3 weeks old cow urine baits [5]. All odors were placed on the ground about 30 cm upwind of the trap. The poles of traps were greased to prevent fly predators, mainly ants. Traps were allowed to stay at the site of deployment for a period of 48 h before collection. After 48 h of deployment, the catchments of each trap were sorted by fly species and then counted, identified and analyzed. The apparent density of the tsetse flies was calculated as the number of tsetse fly catch/trap/day (FTD) [6]. Sex of all collected flies was identified by observing the posterior end of the ventral aspect of the abdomen by hand lens and stereomicroscope hence male flies were identified by enlarged hypopygium in the posterior ventral part of the abdomen which is absent in female flies.

### Fly dissection

The dissection procedure was carried out as described by the FAO Training manual for tsetse control personnel who began by removing wings and legs after wing fry analysis was performed the age of collected male flies was recorded and ovary analysis was used to determine the age of female flies similarly. Then, freshly killed tsetse flies were dissected under a dissecting microscope by using 0.9% normal saline. Trypanosome infections in dissected body parts of tsetse flies (i.e. midgut, salivary gland and mouthpart or proboscis) were observed using a compound microscope at a magnification of ×40 times [7]. Parasites detected in the midgut, salivary glands, and mouthparts (proboscis) were considered as Trypanozoon (*T. brucei*), those detected in the mouthparts (proboscis) and midguts were Nanomonas (*T. congolense*), and those found in the mouthparts (proboscis) only was considered in the group of Duttonella (*T. vivax*), immature infections considered when only found in the midgut. Finally, Giemsa stained smears (slides) were examined under oil immersion compound microscope (100 times magnification) for trypanosome species identification based on their morphological appearances [8, 13, 14]. The Infection rate of the parasite (IR) was calculated using the following formula [7]:

$$\text{Infection rate }\left( {\text{IR}} \right) = \frac{{{\text{Number of tsetse flies infected }} \times 100}}{\text{Total number of dissected flies}}.$$

### Data analysis

Data collected from each deployed trap were coded into appropriate variables and entered in Microsoft excel, 2010 spreadsheet. All statically analyses were performed using STATA- 12 software. The Infection Rate (IR) was calculated for all data as the number of infected individuals divided by the number of individuals sampled times 100. Categorical data were analyzed by using the Chi square (c2) test of independence. In all cases, 95% confidence intervals were used and a p-value less than 0.05 were considered significant [9].

## Results

A total of 623 tsetse flies were caught from 70 deployed mono conical traps during the study period. The apparent fly density was found to be 4.45 Flies/Trap/Day for *G. tachinoides* with peak infestation was resulted in Busase Peasant Association (FTD = 1.85) and low (FTD = 0.41) in Adis limat (Table 1).

**Table 1:** Fly composition and distribution in different peasant associations

Peasant associations	River basin	No. of deployed trap	Number of a fly caught			F.T.D
			*G. tachinoides*			
			Male	Female	Total	
Adis limat	Dedessa	15	8	49	57	0.41
Burqa gudina	Dedessa	15	15	78	93	0.67
Gale jimate	Kutala	20	41	173	214	1.52
Busase	Dedessa	20	63	196	259	1.85
Total		70	127	496	623	4.45

From the total of (n = 284) dissected *Glossina tachinoides* flies only (n = 5) positive for Trypanosome resulting in 1.76% Infection Rate. Peak trypanosome infections were observed in female tsetse 1.41%, n = 4 and 0.35%, n = 1 in males (Table 2). Generally, 1.06% of *Glossina tachinoides* were infected by *Trypanosome vivax* and the remaining 0.70% was *Trypanosome congolense*. There was a strong difference between age-related effects in the number of trypanosome infections with all of the infected flies were older than 21 days when compared to those aged less than 20 days *p *=* 0.001*. There was also a strongly significant difference *p *=* 0.001* between hunger stages which indicated that there was no infection of trypanosomes in teneral flies or stage 4 as more stage 2 (replete stage) and stage 1 (gorged) flies were highly susceptible.

**Table 2:** The infection rate of trypanosomes in *Glossina tachinoides* between sex, age, and hunger stage

Risk factors	No. of fly dissected	Trypanosome species found			Infection Rate IR (%)	P-value
		*T*. *congolonse*	*T*. *vivax*	Total infected		
Sex						
Male	88	0	1	1	1.14	0.005
Female	196	2	2	4	2.04	
Total	284	2	3	5	1.76	
Age						
Young (< 20 days)	172	0	0	0	0	0.005
Old (> 21 days)	112	2	3	5	4.46	
Total	284	2	3	5	1.76	
Hunger stage						
Stage 4 (teneral or hungry)	52	0	0	0	0	0.001
Stage 3 (intermediate)	98	0	0	0	0	
Stage 2 (replete)	109	2	1	3	2.75	
Stage 1 (gorged)	25	0	2	2	8.00	
Total	284	2	3	5	1.76	
Overall	284	2	3	5	1.76	

p- value < 0.05, considered as significant

## Discussion

The study was conducted to estimate the infection rate of trypanosome in *Glossina tachinoides*, their distribution and involved parasite species in Limmu Kosa District of Jimma zone which helps to implement appropriate methods for control and suppression of the disease and its vector at the study area.

The study result FTD = 4.45 was in agreement with Vreysen et al. [10], who discussed the distribution and abundance of tsetse flies to be closely associated with ecology, vegetation and habitat factors. Moreover, it is also comparable with different similar results in different study areas FTD = 5.58 in Sokoru District [11] and lower than that of FTD = 10.9 Botor Tollay District, of Jimma Zone as result of variability in ecology, vegetation, and habitat factors [12]. Apart from this fact, there are other findings significantly vary from this result in some Southern Regions of Darmallo District FTD = 23 [13]. There were some implemented control interventions by local farmers by burning and clearing bushes to minimize the impact and magnitude of the vector before the study was conducted, which may be directly related to resulted lower fly density comparative to previous studies.

The overall trypanosome infection rate of 1.76% is not in agreement with Desta et al. 2013 [14], 7.5% due to species involved *G. morsitans sub mor*. According to Bourn, Shaw and Torr [15], relatively low fly infection rate of 1.76% in *G. tachinoides* in present study may be due to least tsetse challenge, which could be explained by the less fly-animal contact at study area and trypanosome-binding lectin proteins (D+ glucosamine and D+ galactosamine) which makes *G. tachinoides* (riverine group) relatively resistant for infection of trypanosome than *G. morsitans sub mor* (Palpallis group). The reason for a comparatively higher infection rate IR = 2.04% in females might be due to their better life expectancy and lower infection rate found in male flies IR = 1.14% can be explained by the low average age of trapped male flies (20 days or less) [14].

According to Adams et al. [16], *T. vivax* is considered to be one of the most important of the Salivarian trypanosomes because of its pathogenicity to cattle and its relatively higher infection rate in *G. tachinoides* and other tsetse fly species which was completely supported by the present findings.

## Conclusion

The presence of *G. tachinoides* with high-density FTD = 4.45 and 1.76% Infection Rate of Trypanosome recommend control and suppression of the vector and parasite was mandatory due to Pathogenic Animal Trypanosomosis.

## Limitation

The study couldn’t include blood-feeding preference of involved vector (G. *tachinoides*) due to lack of PCR (Polymerase Chain Reaction) and molecular test.

## Data Availability

Not applicable in this section however, all required Data will be available on request of correspondent author.

## Abbreviations

FTD: Flies/Trap/Day
IR: Infection rate
*G. tachinoides*: *Glossina tachinoides*
*G. morsitans sub mor*: *Glossina morsitans sub mor*
*T*. *congolonse*: *Trypanosome congolonse*
*T*. *vivax*: *Trypanosome vivax*
*T. brucei*: *Trypanosome brucei*
